# 

**Published:** 1906-06

**Authors:** 


					CONTENTS.
Page
The Relation of the Pelvic Floor to Pelvic Displacements and
Pain in the Female.
?5y JbRNEST W. xlEY LrROVES, M.D., M.b. L,Ond.,
r .K.U.S.
Q7
Lumbaeo.
Bv Charles E. S. Flemming, M.R.C.S., L.R.C.P.
Difficulties in the Surgical Diagnosis and Treatment of Cases
associated with Vomiting in omiaren.
ay JAMES oWAIN, M.b.,
M.D. Lond.. F R.C.S.
n6 I
New Methods of Clinical Pathology.
By I. Walker Hall, M.D.Vict.
Tubal Pregnancy?its Diagnosis and Treatment.
By C. Hamilton
Whiteford, M.R.C.S., i-K.U.F.
126
The New Theorv and Prophylaxis of Puerperal Eclampsia.
By
Eliza L. Walker Dunbar, M.D. Zurich ...
Health Notes in Bristol, 1905.
By D. 5. Davies, M.D. Lond.
*37
Progress of the Medical Sciences:
Medicine.
George Parker. M.D., M.A. Uantab.
140
Sureerv.
Charles A. Morton, F.R.C.S.
145
Obstetrics.
Walter C. Swayne, M.D. Lond.
I5i
Reviews of Books
I57
editorial Notes
i7i
?Notes on Preparations tor the oick
179
'he Library of the Bristol Medico-Chirurgical Society
i85
Meetings of Societies
188
*-?Dituary Notice ...
igx
Local Medical Notes
192

				

